# An Anti-Interleukin-2 Receptor Drug Attenuates T- Helper 1 Lymphocytes-Mediated Inflammation in an Acute Model of Endotoxin-Induced Uveitis

**DOI:** 10.1371/journal.pone.0090216

**Published:** 2014-03-03

**Authors:** Salvador Mérida, María Sancho-Tello, Amparo Navea, Inmaculada Almansa, María Muriach, Francisco Bosch-Morell

**Affiliations:** 1 Instituto de Ciencias Biomédicas, Universidad CEU Cardenal Herrera, Valencia, Spain; 2 Departamento de Patología, Universitat de València, Valencia, Spain; 3 Oftalmología Médica, Fundación para el Fomento de la Investigación Sanitaria y Biomédica de la Comunitat Valenciana, Valencia, Spain; 4 Unidad Predepartamental de Medicina, Universitat Jaume I, Castellón de la Plana, Spain; University of Valencia, Spain

## Abstract

The aim of the present study was to evaluate the anti-inflammatory efficacy of Daclizumab, an anti-interleukin-2 receptor drug, in an experimental uveitis model upon a subcutaneous injection of lipopolysaccharide into Lewis rats, a valuable model for ocular acute inflammatory processes. The integrity of the blood-aqueous barrier was assessed 24 h after endotoxin-induced uveitis by evaluating two parameters: cell count and protein concentration in aqueous humors. The histopathology of all the ocular structures (cornea, lens, sclera, choroid, retina, uvea, and anterior and posterior chambers) was also considered. Enzyme-linked immunosorbent assays of the aqueous humor samples were performed to quantify the levels of the different chemokine and cytokine proteins. Similarly, a biochemical analysis of oxidative stress-related markers was also assessed. The inflammation observed in the anterior chamber of the eyes when Daclizumab was administered with endotoxin was largely prevented since the aqueous humor protein concentration substantially lowered concomitantly with a significant reduction in the uveal and vitreous histopathological grading. Th1 lymphocytes-related cytokines, such as Interleukin-2 and Interferon-γ, also significantly reduced with related anti-oxidant systems recovery. Daclizumab treatment in endotoxin-induced uveitis reduced Th1 lymphocytes-related cytokines, such as Interleukin-2 and Interferon gamma, by about 60–70% and presented a preventive role in endotoxin-induced oxidative stress. This antioxidant protective effect of Daclizumab may be related to several of the observed Daclizumab effects in our study, including IL-6 cytokine regulatory properties and a substantial concomitant drop in INFγ. Concurrently, Daclizumab treatment triggered a significant reduction in both the uveal histopathological grading and protein concentration in aqueous humors, but not in cellular infiltration.

## Introduction

Uveitis is an ophthalmological disorder that causes vision loss and involves several heterogeneous diseases, all characterized by intraocular inflammation starting firstly in the uvea, whose differently involved immune pathways remain to be accurately described [1]. Ocular inflammation mainly involves the uveal tract but can also extend to other ocular structures such as the retina or vitreous. There are numerous causes involved, including systemic autoimmune disorders and infection.

The commonest form of uveitis is acute anterior uveitis (AAU), which is considered to have a better visual prognosis than other forms of uveitis; AAU represents up to 92% of all cases of uveitis, and therefore contributes to visual loss from uveitis [2]. In the past few years, inflammation has been recognized as a major driving force of AAU. It is now well-established that, starting from the initial lesion to the iris and the aqueous humor in the eye, numerous cellular and molecular inflammatory components participate in the disease process. Monocyte-derived macrophages and T-lymphocytes are the predominant invading immune cells found in evolving lesions. Both cell types produce a wide array of soluble inflammatory mediators (cytokines and chemokines) that are critically important in the initiation and perpetuation of the disease [3].

Uveitis represents a wide spectrum of intraocular inflammatory conditions and includes various autoimmune and infectious etiologies. Endotoxin-induced uveitis (EIU) is a useful model of human anterior uveitis that is not autoimmune. It has served as a valuable model for ocular acute inflammatory processes driven by innate immune mechanisms and their effects on the tissue, elicited by systemic injection of bacterial endotoxin [4]. So, it is an acute form of uveitis that can be induced by giving rats systemic injections of a sublethal dose of lipopolysaccharide (LPS), a component of the cell walls of Gram-negative bacteria [5]. EIU is marked by the vasodilatation of the iris and vascular changes in the ciliary body, accompanied by increased vascular permeability and a breakdown of the blood-aqueous barrier [6], [7]. In the anterior segment of the eye, it induces a disruption of the blood-barrier that triggers protein leakage in the anterior chamber by the infiltration of macrophages and neutrophils into the eye. Inflammation ensues 4 h after the LPS injection, peaks after 24-48 h, and declines 96 h after disease induction [1], [7].

Wide clinical and experimental evidence support the role of particular Gram-negative bacteria or their lipopolysaccharides (LPS) in the pathogenesis of noninfectious, immune-mediated AAU [7].

In EIU cytokines and chemokines are involved in the response, but why systemic injection of LPS would cause a response in the eye, is still not well known [4]. So, in the development and modulation of this animal model for acute ocular inflammation, various cytokines and chemokines released by infiltrating cells, such as TNF-alpha, INF-gamma, TGF-beta, IL-1, IL-6, IL-8, MCP-1, Rantes and inflammatory mediators are important [1], [8], [9]. Moreover in EIU, Th1 activation seems predominant, but with any kind of Th2 participation [1].

Indeed, in other experimental uveitis models, the inflammatory mediator expression pattern largely paralleled that seen in Th1-induced disease [10]. So, experimental autoimmune uveitis disease induction has been characterized by the polarization of early T-helper (Th)0 or Th2-like responses towards Th1, whereas resistance to disease is associated with both regulatory cells and polarization towards a Th2 pathway [11].

Thus, secretion of cytokines and chemokines, and the LPS activation of neutrophil and mononuclear cells in ocular tissues, trigger the release of proteolytic enzymes and free radicals, among other substances that are related to oxidative stress [12], [13].

In the present work, we were interested in the possible effect of Daclizumab, a humanized mononuclear antibody directed against high-affinity interleukin-2 (IL-2R), on ocular inflammation, more precisely in acute anterior uveitis, by using a well-known EIU model. Daclizumab is a humanized monoclonal antibody of the IgG1 subtype that binds to the Tac epitope on the interleukin-2 (IL-2) receptor α-chain (CD25) and, effectively blocks the formation of the high-affinity IL-2 receptor [14]. Interaction of IL-2 with this receptor is required for the clonal expansion and continued viability of activated T-cells, which means that so, IL-2Rα is expressed on activated T cells, but not on resting T-cells or B-cells, NK cells and monocytes. Indeed, IL-2 is a cytokine produced by and acting on T-lymphocytes with complex actions. So, IL-2 augments the immune responses mediated by conventional T-cells [14], [15]. On the one hand, CD4+CD25+FoxP3+ regulatory T-cells (Tregs) depend on IL-2, so IL-2 is critical for peripheral tolerance. On the other hand, IL-2 augments the immune responses mediated by conventional T-cells [14], [15].

Since in 1999 Nussenblatt et al. [16] studied Daclizumab for treatment of noninfectious intermediate and posterior uveitis, it has been also studied for the treatment of autoimmune uveitis [17] and recently, it has been securely and effectively used for the treatment of intermediate and posterior uveitis in adults [18], [19]. Furthermore, high-dose intravenous Daclizumab can help reduce active inflammation in active Juvenile Idiopathic Arthritis-associated anterior uveitis, but patients need to be monitored for potential side effects [20].

Daclizumab has been also shown to be effective in reducing acute rejection episodes in patients undergoing different solid organ transplants, including renal [21], lung [22] and pancreatic [23]. Daclizumab administration has also led to a marked and prolonged decrease in Tregs in cancer patients, suggesting that Daclizumab may be an effective and available therapeutic agent for Tregs modulation in these patients [24].

Thus, the purpose of the present study is to investigate the efficacy of Daclizumab in an animal model of acute anterior ocular inflammation that is not autoimmune. We investigated the effects of Daclizumab on cellular infiltration, protein leakage, oxidative stress markers, and also on the levels of cytokines and chemokines in the aqueous humor of rats in an attempt to elucidate the potential beneficial effects of Daclizumab in acute anterior uveitis.

## Materials and Methods

### Animal manipulation

Male Lewis rats weighing 250–300 g were used (Harlan Ibérica SL, Barcelona, Spain). All the animal manipulations were carried out according to European Union (86/608/EEC) and ARVO (Association for Research in Vision and Ophthalmology) international regulations. The study was also approved by the Committee on the Ethics of Animal Experiments of the Universidad CEU-Cardenal Herrera (Permit number: 315/2006). Animals were individually caged and maintained in a 12 h/12 h light/dark environment of less than 100 cds/m^2^ illuminance during the light phase, with controlled temperature (20°C) and relative humidity (60%), and access to food and water *ad libitum*.

### Animal treatment

Rats were anesthetized with an intraperitoneal injection of ketamine (100 mg/kg body weight) and azepromazine (2.5 mg/kg body weight) before any treatment, and were randomly assigned to four different experimental groups.

Endotoxin-induced uveitis was induced by footpad injections of 200 µg LPS (100 µg each footpad) from *Salmonella typhimurium* (Sigma-Aldrich, St. Louis, MO, USA), diluted in 0.2 ml saline. Control animals received the same amount of saline without LPS [25].

Immediately after the LPS injection, rats were i.v. injected with Daclizumab (5 mg/kg body weight) diluted in saline, or the same amount of saline without Daclizumab. Table 1 summarizes the different treatments and the number of rats used.

**Table 1:** Summary of treatment groups.

Footpad injection	i.p. injection	Treatment group	No. of animals	
Saline	Saline	Control (C)		13
Saline	Daclizumab	Daclizumab (D)		13
Endotoxin	Saline	Endotoxin (E)		18
Endotoxin	Daclizumab	Endotoxin+Daclizumab (E+D)		15

### Sacrificing animals

Twenty-four hours after LPS and/or Daclizumab treatment, rats were anesthetized and sacrificed by cervical dislocation. Then, the aqueous humor was obtained and enucleation was carried out immediately. Eyes were either stored in 10% buffered formaldehyde for histopathological evaluation or frozen at −80°C until processed for the biochemical analysis.

### Aqueous humor collection and cell counting

Immediately after sacrificing the animals, aqueous humors were collected from both eyes by an anterior chamber puncture using an insulin syringe. Approximately, 20 µl/rat were obtained.

The integrity of the blood-aqueous barrier was assessed 24 h after the uveitis induction by evaluating two parameters: cell count and protein concentration in aqueous humors. For cell counting, an aliquot of aqueous humor was diluted in an equal volume of trypan blue, and cells were counted with a hemocytometer under a light microscope. The number of viable and non- viable cells per field (equivalent to 0.1 µl) was manually counted, while the number of cells per microliter was obtained by averaging the results of at least four fields from each sample.

After performing the cell count, aqueous humors were centrifuged at 300*xg* for 5 min; the cell-free supernatant was removed and frozen at −80°C until use.

### Histopathological evaluation

Eyes were stored in buffered paraformaldehyde for 24–48 h to be then embedded in paraffin following standard procedures. Sagittal sections (3-µm thick) were cut near the optic nerve head, and were stained with hematoxylin and eosin (HE). The histopathology of all the ocular structures (cornea, lens, sclera, choroid, retina, uvea, and anterior and posterior chambers) was evaluated. The histopathological evaluation of inflammation was scored with grades 0–3, these being: no infiltrating cells (0), mild (1), moderate (2) or severe (3) cell infiltration.

### Biochemical analysis

Lense-less eyes were removed from enucleated eyes given their high glutathione concentration. On the one hand, Glutathione (gamma-glutamyl-cysteinyl-glycine, GSH) is the most abundant low-molecular-weight thiol, and GSH/glutathione disulfide is the major redox couple in animal cells. On the other hand, Malondialdehyde (MDH) is one of the most prevalent byproducts of lipid peroxidation during oxidative stress. So, lense-less eyes were frozen at −80°C until processed for the biochemical analysis of oxidative stress markers: malondialdehyde (MDA) and glutathione (GSH) concentrations, glutathione peroxidase (GSH-Px) activity, and protein concentration.

Eyes were homogenized in 0.2 M potassium phosphate buffer, pH 7.0, and were frozen at −20°C until use.

The protein concentration was measured according to a modified method described by Lowry et al. [26], [27].

Cytokines and chemokines become redundant secreted proteins with growth, differentiation, and activation functions that regulate and determine the nature of immune responses and control immune cell trafficking and the cellular arrangement of immune organs. The enzyme-linked immunosorbent assays (ELISA) of the aqueous humor samples were performed to quantify the levels of the different chemokine proteins (MIP-1α, MIP-2, MIP-3α, GRO/KC, RANTES, MCP-1, Fractalkine) and cytokine proteins (IL-1α, IL-1β, IL-2, IL-4, IL-6, IL-10, TNFα, INFγ, GM-CSF). ELISAs (Searchlight Multiplex rat Assays; Pierce Biotechnology Inc., Woburn, MS, USA) were carried out following the manufacturer's instructions.

MDA was measured in eye homogenates by high-pressure liquid chromatography (HPLC, Waters-LC Module I Plus, Waters Cromatografia, SA, Spain), following the Richard's procedure modified by Romero [28], [29]. HPLC was done in a Spheryc-5, ODS 5 µm, 250×4.6 mm column (Brownlee-Columns), at a flow rate of 1 ml/min [29]. Values are expressed as nmol/mg of protein.

GSH was also measured in eye homogenates by HPLC (Gilson International B.V. Spain) following the procedure of Reed *et al*. [30]. A 3-Spherisorb NH2 5 µm, 250×4.6 mm column (Waters Cromatografia, SA, Spain) was used at a flow rate of 1 ml/min. Values are expressed as nmol/mg of protein.

Glutathione peroxidase activity was measured spectrophotometrically by monitoring NADPH oxidation at 340 nm, as described Paglia and Valentine [31]. Values are expressed as nmol of NADPH oxidized/min/mg of protein.

### Statistical analysis

Data are expressed as the mean ± SE. Comparisons between groups were made by one-way ANOVA. We performed the analysis of variance of the data obtained by the Levene test, using the LSD test as a *post hoc* test when the data showed homogeneity in their variances (*p*<0.05), or a Dunnet T3 test when variances differed. Statistical differences were set at the *p*≤0.05 level.

No statistically significant differences were found between the data obtained from the Control and Daclizumab groups for any of the parameters studied.

## Results

### Effect of Daclizumab on protein concentration and cellular infiltration in aqueous humors

**Figure 1:**
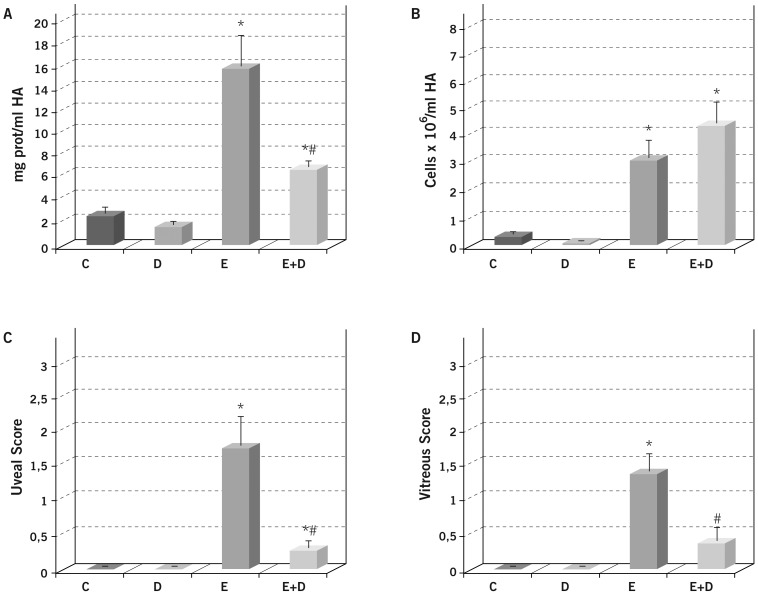
Effect of Daclizumab on protein concentration (A) and cellular infiltration (B) in the aqueous humor collected 24 h after endotoxin treatment, and on the histopathological score of uveal (C) and vitreous (D) cellular infiltration. Each value represents the mean ± SE. ^*^ *p*≤0.05 *vs*. the Control and Daclizumab groups and ^#^ *p*≤0.05 *vs.* the Endotoxin group.

Severe inflammation was noted in the rat anterior chamber of the eye at 24 h post-endotoxin administration, suggesting a disruption of blood-barrier integrity. Thus, a significant increase in protein concentration (*p*≤0.05 *vs.* the C and D groups; Figure 1A) and in inflammatory cellular infiltration (*p*≤0.05 *vs.* the C and D groups; Figure 1B) was observed in the aqueous humors of endotoxin-treated rats.

Administering Daclizumab along with endotoxin moderately prevented the inflammation observed in the anterior chamber of the eyes since a significant reduction was observed in the aqueous humor protein concentration (*p*≤0.05 *vs.* the C, D and E groups), but not in cellular infiltration (*p*>0.05 *vs.* the E group; Figure 1A, B).

### Histopathological findings

**Figure 2:**
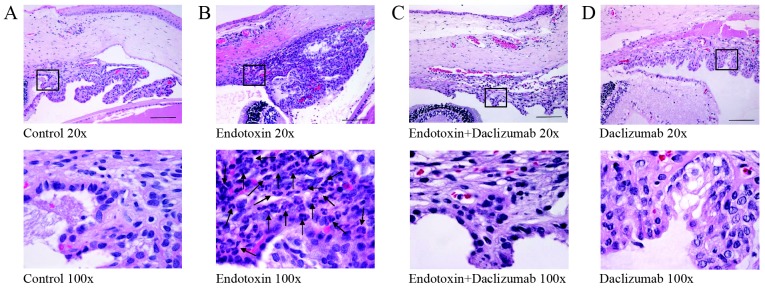
Histopathological changes in the anterior segment 24/or endotoxin treatment, stained with HE. Numerous inflammatory cells (arrows) were observed to infiltrate the extravascular uveal tissue in the endotoxin-treated eyes (B), whereas a significant reduction was observed when Daclizumab was also injected (C). No inflammatory cells were observed in either the Control (A) or Daclizumab (D) groups. Bars represent 250 µm.

Uveitis was achieved by endotoxin treatment, as demonstrated by the significant increase in the number of inflammatory cells in the uveal tissue (*p*≤0.05 *vs.* the C and D groups; Figure 1C) observed in the histopathological sections (Figure 2). The simultaneous Daclizumab administration largely prevented endotoxin-induced uveitis, with a significant reduction noted in the uveal histopathological grading (*p*≤0.05 *vs.* the E group).

Endotoxin treatment also brought about a significant increase in inflammatory cells in the posterior chamber (*p*≤0.05 *vs.* the C and D groups; Figure 1D), although it was less severe than in the uveal tissue. Similarly, the simultaneous administration of Daclizumab and endotoxin largely prevented endotoxin-induced vitreous inflammation (*p*≤0.05 *vs.* the E group).

Retinal tissue showed no morphological alterations at 24 h post-systemic endotoxin administration, and no other histopathological alteration was observed in the other ocular tissues (cornea, lens, choroid, or optic nerve) in any of the groups studied.

### Daclizumab prevents endotoxin-induced oxidative stress

**Figure 3:**
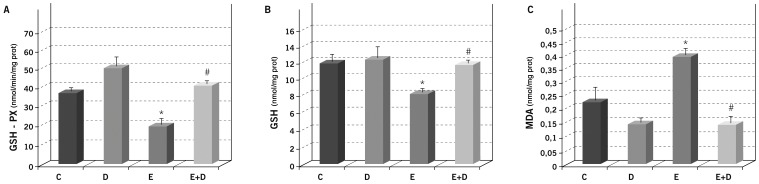
Effect of Daclizumab on endotoxin-induced oxidative stress in rat eyes. Glutathione peroxidase activity (A), glutathione (B) and malondialdehyde (C) concentrations were measured in the eye homogenates of the different study groups. Each value represents the mean ± SE. ^*^ *p*≤0.05 *vs.* the Control and Daclizumab groups, and ^#^ *p*≤0.05 the Endotoxin group.

Different parameters relating to oxidative stress were measured in the eye homogenates from rats 24 h after endotoxin and/or Daclizumab treatment. Endotoxin led to a significant reduction in anti-oxidant systems, such as GSH peroxidase and GSH (*p*≤0.05 *vs.* the C and D groups; Figures 3A, B), which fully recovered by a simultaneous Daclizumab administration (*p*≤0.05 *vs.* E group, and *p*>0.05 *vs.* the C and D groups) for both the parameters studied.

Similarly, endotoxin induced a 2-fold significant increase in lipid peroxidation MDA (*p*≤0.05 *vs.* the C and D groups; Figure 3C), whereas simultaneous Daclizumab administration prevented increased lipid peroxidation because the MDA concentration returned to the control values (*p*≤0.05 *vs.* the E group, and *p*>0.05 *vs.* the C and D groups).

### Daclizumab lessens Th1 lymphocytes-related mediators and increases IL-6 cytokine values after endotoxin treatment

Systemic endotoxin treatment induced significant increases in the aqueous humor levels of a wide variety of inflammatory mediators, including all the cytokines measured (*p*≤0.05 *vs.* the C and D groups; Table 2), with increases of between 3- and 500-fold in the control values. However, the simultaneous endotoxin and Daclizumab administration did not significantly vary most of cytokine values in relation to endotoxin group ones (*p*>0.05 *vs.* the E Group in Table 2).

**Table 2:** Cytokine concentration in the aqueous humors of the different study groups.

	Control	Daclizumab	Endotoxin	Endotoxin+Daclizumab
IL-1a	35.1±5.1	33.4±8.9	76.3±18.8^*^	74.6±10.2^*^
IL-1b	4.9±0.9	5.3±2.1	918.6±243.4^*^	1319.3±313.9^*^
IL-2	9.8±0.3	9.7±0.6	731.6±213.2^*^	245.3±99.3^*#^
IL-4	94.1±2.1	93.6±3.8	293.6±35.6^*^	323.3±47.1^*^
IL-6	10.4±1.2	12.8±5.9	634.3±260.2^*^	6085.1±1233.6^*#^
IL-10	4.1±1.0	3.7±2.9	102.2±20.9^*^	80.7±13.4^*^
TNF-a	7.5±2.8	7.2±1.5	151.8±24.1^*^	213.7±47.5^*^
INF-γ	11.6±0.5	11.9±1.0	99.3±14.3^*^	28.1±10.8^#^
GM-CSF	154.9±19.2	162.0±23.1	1097.3±287.6^*^	1213.2±381.3^*^

All the values are expressed as the mean ± SE, and are expressed in pg/ml of aqueous humor, ^*^ *p*≤0.05 *vs*. C and D; ^#^ *p*≤0.05 E+D *vs*. E.

Interestingly, Th1 lymphocytes-related cytokines, such as IL-2 (Interleukin-2) and IFN-γ (Interferon-γ), which increased the control values after endotoxin administration by between 8- and 76-fold (Table 2), presented a significant reduction when Daclizumab was administered along with endotoxin (*p*≤0.05 *vs.* E Group), thus preventing the aforementioned endotoxin-induced increases. Furthermore, the cytokines related to monocytes (IL-1β, IL-6, TNF-α) displayed -all of them- a tendency to increase when Daclizumab was administered along with endotoxin. Interestingly, IL-6 cytokine showed a significant and great increase (*p*≤0.05 *vs.* E Group) in these conditions. Nevertheless, the cytokines related to Th2 lymphocytes (IL-4, IL-10) did not significantly alter when Daclizumab was administered along with endotoxin (*p*>0.05 *vs.* E Group; Table 2).

### Daclizumab increases some of the macrophages related chemokines after LPS induction

Similarly to cytokines, endotoxin treatment induced significant increases in the concentration of all the chemokines studied in aqueous humors (*p*≤0.05 *vs.* the C and E groups; Table 3), with increases of between 20- and 3000-fold in the control values. The effect of Daclizumab on endotoxin-stimulated chemokines was heterogeneous, so it induced no significant changes in most of the chemokines studied in relation to the E group (*p*>0.05 *vs.* E Group; Table 3). However, some related macrophages chemokines, such as MIP-2, MIP-3α and GRO/KC, displayed significant increases (*p*≤0.05 *vs.* E Group; Table 3).

**Table 3:** Chemokine concentration in the aqueous humors of the different study groups.

	Control	Daclizumab	Endotoxin	Endotoxin+Daclizumab
MIP-1a	1.2±1.1	1.5±0.4	1131.4±181.1^*^	1225.3±211.2^*^
MIP-2	13.8±2.7	12.3±12.8	376.4±114.2^*^	1202.1±168.5^*#^
MIP-3a	3.8±3.1	3.1±2.7	9898.6±2257.6^*^	23344.6±4318.9^*#^
GRO/KC	68.2±12.4	58.3±29.7	4981.9±933.1^*^	14641.5±3268.7^*#^
RANTES	6.9±2.2	5.3±1.7	967.4±392.5^*^	912.8±341.6^*^
MCP-1	118.7±44.1	109.2±21.3	123923.7±43862.3^*^	152662.3±53798.8^*^
Fractalkine	49.4±11.3	36.1±16.3	714.8±97.3^*^	819.2±189.3^*^

All the values are expressed as the mean ± SE, and are expressed in pg/ml of aqueous humor, ^*^ *p*≤0.05 *vs*. C and D; ^#^ *p*≤0.05 E+D *vs*. E.

## Discussion

Diverse studies have explored varied cytokine profiles in different forms of uveitis, including EIU [10], [11]. So, there are numerous data which indicate that cytokines and chemokines play a crucial role in the uveitis development, which not only regulate the nature of immune responses [11], [32], but have been detected in ocular fluids or tissues in inflamed eyes reaching a maximum 24 hours after LPS injection [33]. Thus, an elevated expression of cytokines, such as tumor necrosis factor alpha (TNFα), interferon-γ (INFγ), interleukin-6 (IL-6), Interleukin-1 (IL-1) and Interleukin-2 (IL-2), has been observed concomitantly with maximum EIU [9], [34]–[35]. Moreover, Th1 activation seems to be predominant in EIU model, but with concomitant Th2 participation [1].

Our results are consistent with these previous studies because we found several overexpressed cytokines and chemokines in our experimental uveitis model (Tables 2 and 3). Similarly, they indicate that cellular infiltration and protein leakage into the anterior chamber of rat eyes reach high levels at 24 h after LPS injection. Thus, we found that Th1 lymphocytes-related cytokines (IFNγ, IL-2) increased in endotoxin-treated animals when compared to control ones. Besides, the cytokines related to other inflammatory mediators, such as the Th2 lymphocytes ones (IL-4, IL-6, IL-10), or the macrophages ones (IL-1β, IL-6, TNF-α) also exhibited high levels in them.

Interleukin-2 is a pleiotropic cytokine that drives T-cell growth, augments NK cytolytic activity, induces the differentiation of Tregs and mediates activation-induced cell death. It's known that Interleukin-2 plays an important role in the regulation of the expression of IL-2 receptors and the synthesis of IFNγ by T-lymphocytes. Hence, previous studies have indicated that IL-2 can promote IFNγ production [36] and that the production of IFNγ expression is cell-cycle dependent [37].

Daclizumab prevents binding of IL-2 to its high-affinity receptor and subsequent signaling. McDyer et al. [38] suggested that it has a major effect on IFNγ production by T-cells. Specifically, Daclizumab inhibited the IFNγ production by activated human peripheral blood mononuclear cells through its impact on both the IL-12-dependent and IL-12- independent pathways. Furthermore, it has been suggested that the inhibitory activity of Daclizumab is probably due to a direct effect of IL-2 on CD40L expression [39], a protein member of the TNF superfamily molecules that is primarily expressed in activated T-cells. So CD40L/CD40 interactions on antigen-presenting cells play an important role in promoting T-cell activation and Th1 differentiation. CD40L is also expressed at a higher frequency and for a sustained period in polarized Th1 CD4^+^T-cell cultures in relation to their Th2 counterpart [39].

IL-2 also interferes with IL-6-dependent signaling events, including downregulation of expression of the IL-6 receptor [40].

All in all, our results are consistent with these previous studies. So, Daclizumab administration along with endotoxin reduced the Th1 lymphocytes-related cytokines levels, such as IL-2 and IFNγ, by approximately 60–70%. However, the cytokines related to Th2 lymphocytes (IL-4, IL-10) maintained high levels when Daclizumab was administered along with endotoxin. On the other hand, some of the cytokines related to monocytes and macrophages, such as TNFα, IL-6, IL-1α and IL-1β maintained high concentration levels under the same conditions and even exhibited an increasing tendency in their values (*p*>0.05 *vs.* E Group). Moreover, one of them, IL-6, showed a great and significant increase (*p*≤0.05 *vs.* E Group).

Oxidative stress is also suggested to be pathogenic in inducing inflammation in the eye [41]. Hence, the presence of reactive oxygen species (ROS) exerts their toxic effects on neighboring tissues, which involves an important event for the perpetuation of intraocular inflammation, and simultaneously weakens the tissue's own antioxidant defense system.

ROS react non-specifically and rapidly with biomolecules, including DNA, proteins, lipids and carbohydrates, and various research works have elucidated roles for ROS in causing molecular damage, such as DNA mutations, lipid peroxidation and protein oxidation [41]–[43].

In the present study, we have measured the different parameters related to oxidative stress in eye homogenates from rats 24 h after endotoxin and Daclizumab treatment. Administration of Daclizumab markedly increased intracellular glutathione (GSH), an abundant natural thiol antioxidant and co-substrate for detoxification enzymes like glutathione peroxidase (GSH-Px). Simultaneously, it also increased the levels of GSH-Px, a selenium-containing enzyme that reacts with GSH molecules to regulate lipid peroxidation. Finally, increased endotoxin-induced lipid peroxidation was also shown by the malonyldialdehyde concentration levels, a product of fatty acid peroxidation. Lipid peroxidation products such as MDA may inhibit different enzymes like GSH-Px in a concentration-dependent manner [44], [45]. Thus, after endotoxin and Daclizumab treatment, high MDA concentration levels from endotoxin-induced rat eyes returned to the control values. This antioxidant protective effect of Daclizumab may be attributed to several of the observed Daclizumab effects in our study. So and firstly, several studies have shown that IL-6 is redox-regulated and can maintain a protective function in organs subjected to oxidative stress. IL-6 may induce apoptosis of neutrophils and hence contribute to neutrophil clearance [46]. Acute inflammation is characterized by an initial infiltration of neutrophils, which is then replaced by monocytes and T cells in order to prevent increased tissue damage from the accumulation of neutrophil-secreted proteases and reactive oxygen species at the site of inflammation. Upon infiltration to the site of inflammation, neutrophils produce soluble IL6 receptor (sIL6R) by proteolytic cleavage of the membrane bound form (mIL6R). Proteolytic processing of the IL-6R from invading neutrophils subsequently may drive IL-6 transsignaling in resident tissue cells, leading to a switch from neutrophil to monocyte recruitment by enhancing mainly monocyte-attracting chemokines. IL-2 down regulates the expression of the IL-6 receptor [40]. So it's feasible than Daclizumab upregulates IL-6 receptor expression. And, in this way, during acute inflammation, the high level observed of IL-6 cytokine after Daclizumab treatment may favor the resolution of the neutrophilic infiltrate. The finding that IL-6 induces neutrophil apoptosis further supports the notion that IL-6 substantially contributes to the resolution of acute neutrophil infiltration [47]. Besides its role in attracting monocytes, IL-6 transsignaling has been shown to skew monocyte differentiation towards macrophages by upregulating M-CSF receptor expression.

Moreover, recently Wruck et al. [48] have suggested a possible role of IL-6 in oxidative stress defense. Nrf2 is a redox-sensitive transcription factor which provides cytoprotection against electrophilic and oxidative stress and is the most potent activator of antioxidant response element-dependent transcription. These authors showed that Nrf2 is a potent activator of IL-6 gene transcription *in vivo*.

Then again, when Daclizumab was administered along with endotoxin, IFN-γ presented a significant reduction. IFN-γ is the most important trigger for the formation and release of ROS [49]. IFN-γ is of prime importance in the development of oxidative stress for antimicrobial and anti-tumoral defense within the cell-mediated immune response. Thus, IFN-γ is the prototypic example of a positive regulator of nitric oxide production by inducible nitric-oxide synthase [50], [51]. Furthermore, IFNγ is the predominant cytokine implicated in the induction of indoleamine 2,3-dioxygenase, the main inducible and rate-limiting enzyme for the catabolism of the amino acid tryptophan through the kynurenine pathway. The interrelationships between indoleamine 2,3-dioxygenase and nitric-oxide synthase in macrophages or glial cells are well known, as are the potential interactions with neurons by means of *N*-methyl-d-aspartate (NMDA)-receptor-induced nitric oxide formation [52].

Our previous work and that of others [35], [41], [53]–[55] demonstrate that antioxidants may inhibit tissue damage caused by inflammatory reactions through ROS regulation. Therefore, the prevention of several inflammatory diseases, or diseases in which inflammation has been found to play an important role in disease pathogenesis and progression, could effectively be achieved by using antioxidants.

The effect of Daclizumab on endotoxin-stimulated chemokines was heterogeneous, since it induced no significant changes in most of the chemokines studied as compared to the endotoxin group in this acute model. The major role of chemokines is to act as a chemoattractant to guide the migration of cells, which can be produced by endothelial cells in response to cytokines, such as TNF-α and IL-1 [11]. So, high levels were reached in some studied chemokines under the same conditions.

All in all, some related M1 macrophages chemokines (MIP-2, MIP-3α, GRO/KC) displayed relevant increases (*p*≤0.05 *vs.* E Group). MIP-2 is secreted by monocytes and macrophages, and is chemotactic for polymorphonuclear leukocytes and hematopoietic stem cells. MIP-3a is strongly chemotactic for lymphocytes and weakly attracts neutrophils. MIP-3a expression has been observed to be dependent on IL-6 amplifier activation [56]. GRO/KC is expressed by macrophages, neutrophils and epithelial cells and recently some authors [57] have suggested that this chemokine presents any kind of protective role.

Tregs, which constitute approximately 10% of peripheral CD4^+^ T-cells, suppress immune system activation and maintain immune homeostasis and tolerance to self-antigens [58]. Tregs require IL-2 for survival, so they consume, but do not produce, IL-2 and other cytokines that promote growth and/or survival [59]. Furthermore, Tregs promote the induction of alternatively (M2) activated monocytes/macrophages [60]. Additionally, induction of regulatory T-cells by macrophages is dependent on ROS production [61] and IL-6 inhibits TGF-beta-induced Treg differentiation [62]. Consequently in our acute EIU model, it is possible that Daclizumab partially acts as an agent for Treg modulation, so it indirectly and modestly promotes the activity of classical M1 macrophages. Indeed, an increased in the different levels of cytokines and chemokines linked to M1 macrophages activation, such as TNFα (*p*>0.05 *vs.* E Group), MIP-2, MIP-3a, GRO/KC and IL-6 (*p*≤0.05 *vs.* E Group), would be in accordance with this point.

Finally, in addition to the histologic preservation of ocular structures, treatment with Daclizumab is associated with a diminished accumulation of protein in aqueous humors, but not inflammatory cell infiltration, similarly to the manifestations of the beneficial effects of Daclizumab treatment in this inflammation model.

### Conclusions

At 24 h post-administration, Daclizumab treatment in EIU reduced Th1 lymphocytes-related cytokines Interleukin-2 and Interferon gamma by around 60–70% and was seen to play a protective role in endotoxin-induced oxidative stress. It is feasible to believe that this antioxidant protective effect of Daclizumab may be related to several of the observed Daclizumab overcomes in our study, including IL-6 cytokine regulatory assets and significant reduction of INFγ cytokine, a very important trigger for the formation and release of ROS. Daclizumab treatment also significantly reduced the uveal histopathological grading and the protein concentration in aqueous humors, but not the cellular infiltration.
